# A Simple Label-Free Aptamer-Based Electrochemical Biosensor for the Sensitive Detection of C-Reactive Proteins

**DOI:** 10.3390/bios12121180

**Published:** 2022-12-18

**Authors:** Huilin Gao, Yongchang Bai, Baixun He, Cherie S. Tan

**Affiliations:** 1 Academy of Medical Engineering and Translational Medicine, Tianjin University, Tianjin 300072, China; 2 Tianjin Key Laboratory of Brain Science and Neuroengineering, Tianjin 300072, China

**Keywords:** C-reactive protein, DNA aptamer, gold nanoparticles, carboxylated graphene oxide

## Abstract

The level of C-reactive protein (CRP) in the human body is closely associated with cardiovascular diseases and inflammation. In this study, a label-free functionalized aptamer sensor was attached to an electrode trimmed with in-gold nanoparticles and carboxylated graphene oxide (AuNPs/GO-COOH) to achieve sensitive measurements relative to CRP. Gold nanoparticles were selected for this study due to super stability, remarkably high electrical conductivity, and biocompatibility. In addition, carboxylated graphene oxide was utilized to promote the anchorage of inducer molecules and to increase detection accuracies. The sensing signal was recorded using differential pulse voltammetry (DPV), and it produced a conspicuous peak current obtained at approximately −0.4 V. Furthermore, the adapted sensor manifested a broad linear span from 0.001 ng/mL to 100 ng/mL. The results also demonstrated that this aptamer sensor had superior stability, specificity, and reproducibility. This aptamer-based electrochemical sensor has enormous potential in complex application situations with interfering substances.

## 1. Introduction

C-reactive proteins (CRPs) were first revealed in 1930 in the plasma of lung inflammation patients [1]. CRP is a rapid-phase inflammatory protein arising primarily from hepatocytes under the influence of cellular factors such as interleukin (IL)-6 and tumor necrosis element alpha (TNF-α) [2]. As an acute inflammatory mediator in plasma during the inflammation response, CRP plays a regulatory role during inflammation responses by enhancing phagocytosis and activating complement. When the body is exposed to inflammatory stimuli or bacterial infections, the CRP level rises after 4–6 h of activation and increases approximately 1-fold every 8 h, usually peaking at 36–50 h [3]. With the recovery of inflammation, the CRP level declines explosively over 18 to 20 h, approaching the half-life of CRP [4]. It is well-established that CRP concentrations in the body is intimately correlated with heart-related diseases [5,6], cardiovascular diseases [7,8], rheumatoid arthritis [9], cancer [10,11], and inflammation [12]. Recently, the CRP has also been found to be associated with depression [13,14,15,16]. Some studies demonstrated that the epidemiological profile for diagnosing hypo-inflammatory depression can be verified by using the level of C-reactive proteins [17]. Related research also showed that CRP can predict the severity of COVID-19 [18,19,20]. Therefore, it is valuable for detecting CRP accurately.

The current detection methods for CRP include enzyme-linked immunosorbent (ELISA) [21], latex-enhanced immunoturbidimetry [22], single radial immunodiffusion (SRID) [23,24], and immunoscattering turbidimetry [25,26]. However, the limitations of these techniques are also evident: They are vulnerable to interference, time consuming, expensive instruments, and require complex operations, and they are particularly inadequate in the clinical development of effective treatment strategies and timely and rapid CRP detections [27].

The advantages of electrochemical biosensor detection, including its low cost and good sensitivity, selectivity, and reliability, make it an attractive tool for biomarker protein detection [28,29,30,31]. There are a number of electrochemical biosensors that use antibodies as a biometric element for the detection of the CRP [32,33]. In some application cases, however, the peptidase susceptibility and immunogenicity of antibodies may primarily affect the availability of immune sensors [34]. In comparison to antibodies, aptamers are stable, cheap, and well-adapted molecules that are excellent candidates for building blocks in biosensor technology [35,36,37]. In addition, the aptamer conformation is flexible, and the structure is easy to design; these merits make it very promising in electrochemical sensors [38]. Furthermore, in contrast to RNA aptamers, DNA aptamers are more stable and can modify functional clusters, such as biotin, thiol groups, and amino clusters, more efficiently at the 3′- or 5′-end [39]. For this reason, DNA aptamers would be a better-quality recognition layer.

There are several aptamer sensors for the detection of the CRP, such as the systematic evolution of ligands by exponential enrichment by GO; an immobilization-free method for the detection of CRP, which needs to fix the CRP on a solid surface [40]; electrochemical aptamer sensors, which transfer electrons with the help of additional electroactive small molecules [39]; optical aptamer sensors, which perform sample analyses by detecting changes in the optical signal of single or multiple beams in a system [41]; and microfluidic aptamer sensors on integrated microelectromechanical systems microfluidic aptamer sensors, which use the aptamer as a recognition element on a micrometer-scale detection chip [42]. However, these aptamer sensors utilize either chemical or optical labels to enhance the sensing operation, and it would be more convenient to apply a label-free aptamer to detect the CRP based on the change in current signal, resulting from aptamer conformational changes.

Achieving high sensitivity is one of the primary purposes of bioassays [43]. Therefore, in exploring new methods to improve sensitivity, the role of using nanomaterials for signal transmission and amplification is increasingly receiving attention [44]. With excellent electrical conductivity, gold nanoparticles (AuNPs) have been extensively used in biomedical science for many years and can play a role in amplifying analytical signals [45]. Moreover, AuNPs can also immobilize biomolecules and maintain their stability and biological activity [46]. In addition, AuNPs allow direct electronic transfers with the redox protein and the electrode material, which allows electrochemical signaling without the aid of an additional electronic mediator [47]. In particular, electrochemical technology is the most widely applied to metals and nanomaterials in analytical sensing due to its inexpensive and user-friendly characteristics [48]. Consequently, electrochemical techniques promote AuNPs for analytical sensing applications. In turn, AuNPs further contribute to electrochemical sensing due to their excellent electrical conductivity; thus, the combination of the two supplements each other.

Various solid surfaces can be utilized to fabricate aptamer-based sensors. Graphene (Gr) has been determined to be one of the most exploitable substances [49]. Graphene has a single-layer bi-dimensional reticulation of neighboring carbon atoms connected by the sp2 hybridization of outer electrons to form covalent σ-bonds, which can maintain stability and rigid structure [50]. Despite this, graphene also possesses excellent properties, such as great specific surface area and is readily available for surface modification [51]. As an integral derivative of graphene, graphene oxide (GO) not only encompasses the strengths of graphene but also has a significant amount of functional oxygen clusters on the rim and base planes, which makes it a good option for bonding with other molecules [52]. Furthermore, GO can be decorated by hydrophilic groups such as carboxyl groups, allowing good stability, hyper solubility, and dispersibility in aqueous solutions [53]. Many researchers recently used carboxylated graphene oxide (GO-COOH) for electrochemical sensing detections. Li et al. showed the first use of graphene–polyaniline (GR-PANI) composites and GO-COOH to detect estradiol [54]. Upan J. et al. successfully detected alpha-fetoprotein (AFP) by utilizing the observed property that GO-COOH aids in obtaining additional space on the surface and the number of immobilized aptamers [55]. Bharti A. et al. used GO-COOH with gold-platinum bimetallic nanoparticles to analyze the microRNA-21 detection [56].

Within this study, we designed an approach for CRP-sensitive tracking: label-free functionalized electrochemical aptamers were immobilized on gold nanoparticles and carboxylated graphene oxide (AuNPs/GO-COOH) composites to achieve sensitive measurements of the CRP. For label-free sensors, molecular imprinting techniques use the target as a template to synthesize polymer chains, which are anchored to an electrode and change conformations upon binding to the target to enable the label-free sensing of the electrochemical platform [57]. There are also electrochemical aptamer sensors that rely on electroactive small molecules, such as methylene blue (MB), to act as electron transfer agents [58]. Combining thiol groups at the ends of the aptamer, the malachite green aptamers (MGAs) bind to the malachite green (MG) to bring the bis-thiolated aptamer within close proximity to the cantilever’s surface to create higher surface stress [59]. The proposed label-free aptasensor performs conformation changes when it interacts with the target CRP, resulting in an electrochemical response shift. One end of the DNA aptamer was modified with an amino group, which can be bound to GO-COOH with an amide bond to achieve the stable adhesion of the ligand to the electrode surface. Before binding to CRP, the adaptor was perpendicular to the electrode’s surface and was highly flexible. When CRP bound specifically to the aptamer, a rigid aptamer-CRP complex was formed and the aptamer conformation changed, bending toward the electrode’s surface and raising the electrical signature. In this work, we first selected the adaptor sequence of the detectors based on their CRP sequence design. Next, the CRP aptamer with a modified amino group was anchored on the superficial face of the bare electrode trimmed with carboxylated graphene oxide. The CRP aptamer was anchored to the electrode composite via the amide bond and demonstrated high stability, specificity, and reproducibility for CRP. In this study, the morphology and electrochemical feedback of the electrodes were evaluated by scanning electron microscopy and differential pulse voltammetry, respectively.

## 2. Materials and Methods

### 2.1. Apparatus

The compound-based sensing interface was visually characterized via layer-by-layer modifications using a high-resolution scanning electron microscope (SEM, Nova NanoSEM 430, FEI, Columbia, MD, USA). All electrochemical measurements, including square wave voltammetry (SWV), and differential pulse voltammetry (DPV) were performed on a CHI660E electrochemical workstation (ChenHua, Shanghai, China). The experiments were performed using screen-printed electrodes (SPE, poten, Beijing, China) with both working and auxiliary electrodes of carbon and silver/silver chloride as reference electrodes to form a three-electrode system. All electrochemical measurements were carried out at room temperature (25 ± 2 °C). Square wave voltammetry was applied at a scan rate of 100 mV/s with a range of −1.0 V ~ −0.2 V. Differential pulse voltammetry was performed with a voltage range of −0.6 V ~ 0 V, an impulse amplitude of 50 mV, and a pulse width of 50 ms.

### 2.2. Reagents and Materials

Sodium tetrachloroaurate dihydrate (NaAuCl_4_-2H_2_O, Aladdin, Beijing, China), sodium sulfate (Na_2_SO_4_, Aladdin, Beijing, China), carboxylated graphene oxide (GO-COOH, XFNANO, Nanjing, China), phosphate buffer (PBS, Aladdin, Beijing, China), 1-(3-dimethylaminopropyl)-3-ethylcarbodiimide hydrochloride (EDC), N- Hydroxysuccinimide (NHS), MES, sodium chloride (NaCl), bovine serum protein (BSA, Genview, Haosogbio, Tianjin, China), C-reactive protein (CRP) rabbit mAb (ABclonal, Wuhan, China), PSA standard (China Food and Drug Administration), CA125 standard, proplatelet basic protein (PPBP), and B-type brain natriuretic peptide (BNP) were used. CRP aptamer probes were ordered from Synbio Technologies (Suzhou, China). The DNA aptamer nucleotide sequences are as follows:

5′-(NH2) GCCTGTAAGGTGGTCGGT GTGGCGAGTGTGTTAGGAGAGATTGC-3′ [39].

### 2.3. Protocol of Electrochemical Aptamer Sensor

**Figure 1:**
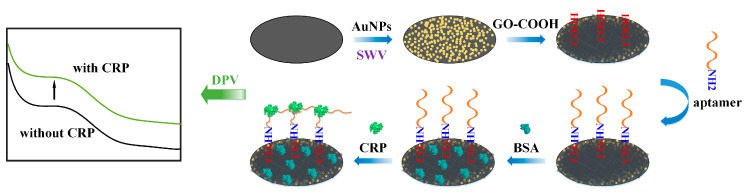
Illustration of the manufacturing process of the label-free C-reactive protein (CRP) aptamer sensor.

Figure 1 illustrates the preparation process of the SPE/AuNPs/GO-COOH aptamer sensor for the electrochemical detection of CRP. The electrodes were rinsed several times with ultrapure water and then blown dry and set aside for operation. The preparation of a 1% mass fraction of NaAuCl_4_-2H_2_O solution and 0.5 M Na_2_SO_4_ nanogold reduction source solution was performed and they were dropwise added on the SPE electrode at a volume ratio of 1:1. The modification of AuNPs continued on the electrode’s surface by using square wave voltammetry with a scanning rate of 100 mV/s at −1.0 V ~ −0.2 V for five turns. It was then rinsed with ultrapure water and air blown until dry. The GO-COOH was decorated on the modified SPE/AuNPs electrode using physisorption at a 2 mg/mL concentration and dried naturally at room temperature. The surface was flushed gently with ultrapure water and then air blown until dry. The CV scan electrode was then used to determine whether GO-COOH was successfully modified relative to the surface. A concentration of 2 mM EDC solution was prepared by mixing 0.4 mg EDC with 1 mL 0.1 M MES and 0.5 M NaCl (pH = 6.0) with shaking. The 0.6 mg NHS was thoroughly mixed with 1 mL PBS to configure the NHS solution with a concentration of 5 mM. The EDC solution and NHS solution were added onto the surface of the modified SPE/AuNPs/GO-COOH electrode dropwise in 1:1 volume and placed at indoor temperature for 15 min to activate carboxyl groups. The modified CRP aptamer was immobilized by amide bonding on the SPE/AuNPs/GO-COOH electrode, which completed carboxyl activation, and then the electrode was placed at room temperature overnight. The SPE/AuNPs/GO-COOH aptamer sensor was closed by adding 1 mg/mL of BSA dropwise to the modified electrode and left for 30 min at 4 °C in a humid environment. At this point, the label-free aptamer sensor modification process had been completed. The specific binding of CRP to the aptamer led to an enhanced current signal as the aptamer changed from a soft structure to a rigid structure, while the orientation shifted from perpendicular to the electrode to curved toward the electrode’s surface, as shown in Figure 1.

### 2.4. CRP Detection

The DPV method was implemented for the electrochemical inspection of SPE/AuNPs/GO-COOH aptamer sensors to establish a relationship between the peak differential pulse current and CRP concentration. The scanning voltage ranged from −0.6 V to 0 V with a pulse amplitude of 50 mV and a pulse width of 50 ms. First, 100 μL of CRP solutions with concentrations of 0.001 ng/mL, 0.01 ng/mL, 0.1 ng/mL, 1 ng/mL, 10 ng/mL, and 100 ng/mL was configured using CRP standards with PBS as the solvent. The gradient concentration of the CRP standard solution was performed for DPV sweeps to establish a relationship between peak differential pulse current and CRP concentrations. To investigate the stability of the sensors that we designed, multiple DPV detections were conducted by using 1 ng/mL of the CRP standard solution on the electrodes on the first, third, fifth, and seventh days to observe changes in the results. The SPE/AuNPs/GO-COOH aptamer sensors were preserved in the 4 °C refrigerator when out of use. The selectivity of the aptamer sensors was further verified by detecting possible disturbing substances with 100 μL of 1 ng/mL PSA, 1 ng/mL of CA125, 1 ng/mL of PPBP, and 1 ng/mL of BNP configured as interfering solutions compared with 1 ng/mL of the CRP standard solution for DPV scale verification. Regarding whether the designed sensor is repeatable, we selected five identical electrodes to test the 1 ng/mL CRP solution for multiple repetitions of the DPV detection.

## 3. Results

### 3.1. Characterization of SPE/AuNPs/GO-COOH Aptamer-Sensing Interfaces

**Figure 2:**
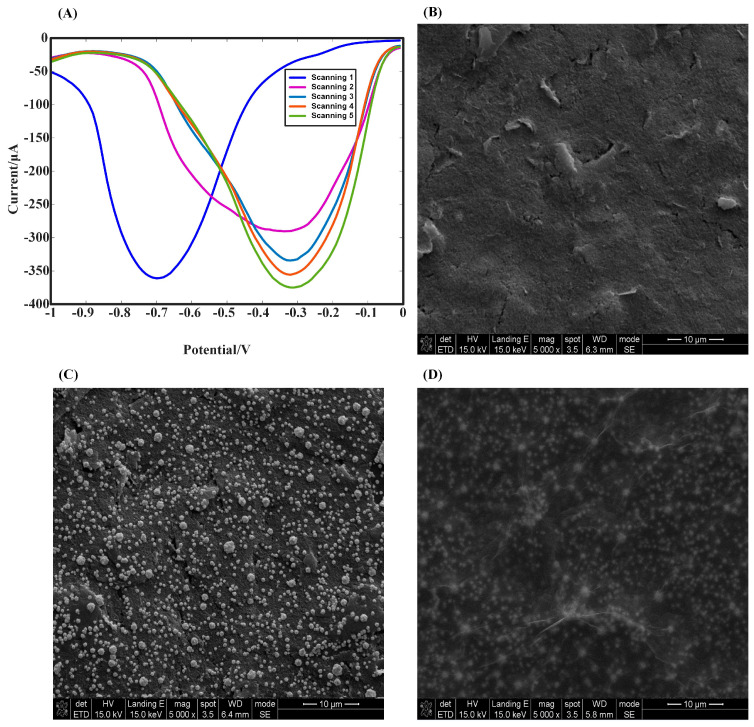
Characterization of the SPE/AuNPs/GO-COOH aptamer’s sensing interface. (**A**) Electrodeposition of AuNPs using the SWV method with five scans. (**B**) SEM electron micrograph of the bare electrode. (**C**) SEM characterization of the electrode surface after the electrodeposition of AuNPs. (**D**) Surface characterization of the GO-COOH physically adsorbed on an electrode containing AuNPs.

We examined the appropriate substances for modifying SPE working electrodes to derive a high electrochemical probe current response. Figure 2A shows the electrodeposition of AuNPs on the electrode’s surface using SWV at a scan rate of 100 mV/s with a scan voltage of −1.0 V ~ −0.2 V. After five scans, extra scans did not enhance the current response significantly, so we performed five SWV scans to deposit AuNPs for the rest of the study. The electrodeposition of gold nanoparticles was achieved by SWV, and the position of the peak appeared at around −0.67 V, implying that AuNPs had been deposited on the electrode’s surface with the scanning process of SWV, completing the SPE/AuNPs electrode structural modification. High-resolution scanning electron microscopy (SEM) was used to characterize the preparation process of the sensors, and the apparent differences in the surface morphology characterization of different substances could be observed. As shown in Figure 2B–D, the morphological changes over the surface at the working electrode of this sensor were analyzed by characterizing the modification process of the electrode at each stage during the preparation of the SPE/AuNPs/GO-COOH-modified electrode with SPE as the substrate. Figure 2B–D show the SEM images of SPE, SPE/AuNPs, and SPE/AuNPs/GO-COOH, respectively. The surface of the bare electrode (Figure 2B) was covered with uneven carbon materials without particle distribution; the SPE/AuNPs (Figure 2C) were derived by depositing AuNPs onto the working electrode surface of the SPE by the SWV method, which showed a significantly enhanced and homogenous particle distribution compared with (Figure 2B), which is consistent with the previous results on the structure and size of gold nanoparticles [60]. Features such as high surface area, low charge transfer resistance, and fast electron transfer rates resulted in AuNPs with enhanced electron transfer velocity. From (Figure 2C), GO-COOH was expected to be a thin film morphology and exhibits wrinkles. When AuNPs and GO-COOH were sequentially revised onto the electrodes, the variation in the surface pattern showed that they could be successfully immobilized onto the electrodes. The layer-by-layer modification provided a sensing platform with excellent stability, selectivity, and reproducibility for adaptors, which together constructed an aptamer sensor with good application performances.

### 3.2. Sensing Detection of CRP

**Figure 3:**
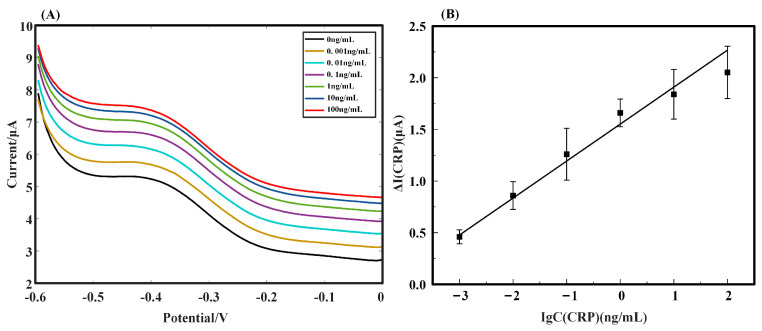
(**A**) Voltage-current diagram of CRP solutions from 0.001 ng/mL to 100 ng/mL using the DPV method. (**B**) The curve of peak currents for each concentration of CRP fitted to the difference between the 0 ng/mL CRP solution and the logarithm of the CRP concentration (n = 3).

The DPV curves of the SPE/AuNPs/GO-COOH aptamer-based sensor for the detection of CRP standard solutions in the concentration range of 0.001 ng/mL ~ 100 ng/mL are shown in Figure 3A. As the concentration of the CRP standard solution progressively increased, the peak DPV current correspondingly went up, obtaining a satisfactory calibration curve in the 0.001 ng/mL ~ 100 ng/mL range. The fitted curves were plotted according to the difference between the peak of the differential pulse current of the detected CRP solutions under various concentrations and the peak of the detected current under the PBS solution ($\Delta I=I-I_{PBS}$). For the logarithm with respect to the concentration of CRP solutions ($\log_{10}C_{CRP}$), as shown in Figure 3B, a current correlation equation is obtained as follows.

(1) $$\Delta I_{CRP}=0.3579\times \log_{10}C_{CRP}+1.5516$$

Linearity ranged from 0.001 ng/mL to 100 ng/mL, with a correlation coefficient of R^2^ = 0.9854. The lower limit of determination was 0.001 ng/mL.

### 3.3. Selectivity, Stability, and Reproducibility Detection of Aptamer Sensors

**Figure 4:**
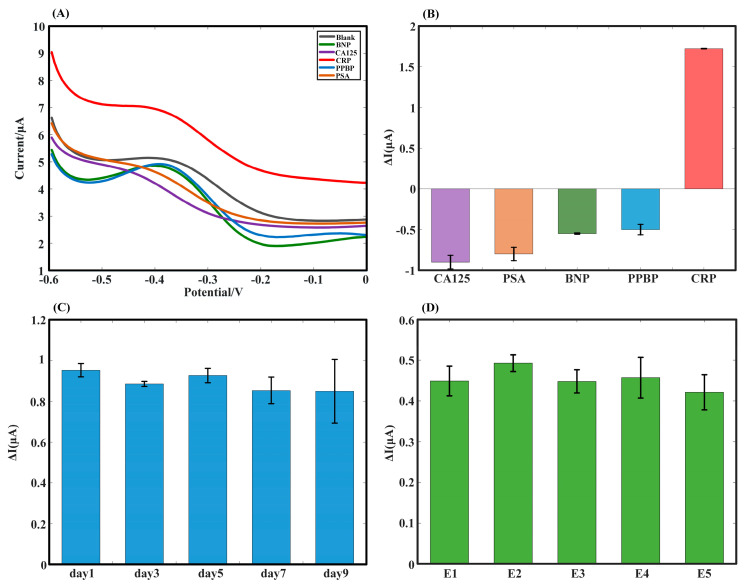
Selectivity, stability, and reproducibility tests based on the SPE/AuNPs/GO-COOH aptamer sensor. (**A**) Voltage-current plot of CRP vs. interfering solutions using the DPV method. Red: 1 ng/mL CRP; black: PBS solution; blue: 1 ng/mL PPBP; green: 1 ng/mL BNP; orange: 1 ng/mL PSA; purple: 1 ng/mL CA125. (**B**) The plot of the selectivity error analysis corresponds to (**A**) (n = 3). (**C**) Error analysis plots were obtained by selecting the electrodes on day 1, day 3, day 5, day 7, and day 9 for testing against 1 ng/mL CRP solution (n = 3). (**D**) Reproducibility test plots of five aptamer-sensing electrodes for multiple assays of 0.001 ng/mL CRP (n = 3).

In this experiment, some possible interfering substances, such as PSA, CA125, PPBP, and BNP, were chosen to evaluate the selectivity of this aptamer sensor. Interference experiments were performed on different electrodes, with one for each piece independently. Selectivity was appraised by recording the signal intensity at 1 ng/mL. DPV was performed for the interfering solutions, with the results shown in Figure 4A. For the current values at −0.4 V, all four interfering solutions, PSA, CA125, PPBP, and BNP, were located below the blank current value, while the DPV scan of the CRP standard solution was greater than the blank current value at −0.4 V. Since the aptamer undergoes conformational changes upon binding to CRP while the other four interfering proteins are physically adsorbed on the electrode, the current will not be enhanced. Moreover, the current values of the disturbed samples were closer to the blank current values at −0.4 V than those of the CRP. This result also reaffirmed that the sensor was enabled to monitor variations in the current precisely via changes in the aptamer’s conformation. The specificity of the developed label-free aptamer sensor was evident from this result. Furthermore, statistical analyses were conducted on the amount of change in the current value at −0.4 V following the DPV scan, which is shown in Figure 4B, where the difference between the interfering substances and PBS currents was negative, and the level of current variation was maintained at a relatively high level. The current response values underwent a significant transformation when the CRP was introduced, with the current becoming positive and the amount of electric current variations being relatively low. These results demonstrated that the aptamer sensor had high selectivity for CRP detections as well as specificity in sophisticated practical scenarios where potential interfering substances were present.

The stability of the aptamer sensor based on SPE/AuNPs/GO-COOH was carried out by keeping the sensing electrode in a refrigerator at 4 °C. Via the experiment, we found that the sensor maintained a period of up to nine days at 4 °C. The sensor was removed and used for the DPV detection of 1 ng/mL CRP on the first, third, fifth, seventh, and ninth days, as shown in Figure 4C. The three replicate experiments in Figure 4C used different separate electrodes. A new electrode was used for each experiment. The slight changes in the current response pointed to the fact that the proposed aptamer sensor retained excellent detection capabilities during long-term storage, demonstrating this sensor’s high stability. The stability DPV scan results based on the SPE/AuNPs/GO-COOH aptamer sensor are illustrated in Figure S1, where it can be seen that the peak results were between 6 and 7 μA for the first, third, fifth, seventh, and ninth days, with the current peaks close to each other. The coefficient of variation is C_v_ (C_v_ = $\frac{\sigma }{\mu })=4\%.$ The superior stability of the ensemble sensor can be confirmed.

An examination of the reproducibility of the SPE/AuNPs/GO-COOH aptamer-based sensor was conducted. Five pieces of aptamer electrodes were arranged in parallel to detect 0.001 ng/mL CRP, while each piece of the electrode was examined repeatedly, as shown in Figure 4D. The current values obtained from the five parallel electrode scans using DPV were compared to the blank control to obtain ΔI. The experiment was repeated three times for each electrode to calculate the standard deviation. In this regard, the five electrodes had close variations in current response ranging from 0.4 to 0.5 μA. This phenomenon represented an acceptable relative deviation of the aptamer sensor during repeated testing with separate electrodes as well as with the same electrode, which collectively demonstrated that the aptamer sensor performed well and was reproducible during CRP detection. The coefficient of variation is C_v_ = 1%. Therefore, this sensor had the potential to demand practical application scenarios with recurring utilizations. These values were not only based on the choice of the modification material for the electrode’s surface but also on the high stability of the DNA aptamer itself and the stability connected to the electrode’s surface by the strong bonding of the amino carboxyl group. The reproducibility of the DPV sweeps based on the SPE/AuNPs/GO-COOH aptamer sensors is shown in Figure S2, and the peak currents are essentially close, reflecting that this modified electrode can maintain accurate results via multiple applications.

### 3.4. CRP Spike Recovery Experiment

In order to investigate the realistic detection capability of this aptamer sensor, CRP standards were added to the artificial serum and diluted to 5 ng/mL, 10 ng/mL, and 20 ng/mL to simulate real samples. The current values were obtained by the DPV, and the results were substituted into the above linear calibration curve to obtain the recoveries and relative standard deviations, as shown in Table 1. The average recovery of 107. 5% and the relative standard deviations were within 10%. The results showed that the sensor was feasible for the determination of CRPs in real samples.

In addition, Table 2 summarizes the comparative analytical performance of the various sensors using the aptamers. We calculated the LOD of this biosensor based on a well-known formula, LOD = 10 × ((3.3 SD-intercept)/slope), which is 2.941 × 10^−4^. It can be seen that the proposed label-free electrochemical sensor offers one of the widest linear ranges compared to other types of sensors, although it does not offer the lowest LOD. This feature gives it an advantage in terms of practical applications.

## 4. Conclusions

A markerless aptamer transducer was developed to detect CRPs using amide bonds to immobilize the aptamer on carboxyl-containing graphene oxide, immobilizing the aptamer on the electrode’s surface. Gold nanoparticles were assigned to amplify the pulse, and carboxylated graphene oxide was used to immobilize the aptamer. SEM was performed to examine the electrode surface modification process. The DPV method was carried out for the sensing detection of the CRP by using this aptamer sensor, exhibiting a wide linear range (0.001 ng/mL to 100 ng/mL). DNA aptamers with amino groups were bound to GO-COOH via amide bonds and oriented perpendicularly to the electrode’s surface. When the aptamer recognized the CRP specifically, the aptamer turned into a rigid structure and was accompanied by a conformational change, that is, bending toward the electrode surface so that the current signal was enhanced. The current signal increased with the accumulation of CRP concentrations within a specific range. Subsequently, the stability and reproducibility of the sensor were studied, and the experimental results proved that the sensor could be stored at 4 °C for more than a week. Moreover, there was a slight variation in the results between different electrodes. In addition, the electrochemical sensor maintained superior characteristics in terms of stability, specificity, and reproducibility. The results indicated that this aptamer sensor could be kept for a certain period and reused repeatedly. The sensor had great potential for complex practical application scenarios where potentially interfering substances were present. Distinct from previous sensors, these label-free aptamer sensors do not require additional electroactive small molecules for electron transport. The aforesaid outcomes reveal that the current variations caused by the aptamer’s conformation alteration can work as sensors with excellent performances. This sensor has excellent specificity and also provides an idea in the field of future sensor development.

## Figures and Tables

**Table 1:** Recovery experiment of CRPs in artificial serum samples.

Samples	Amount Added (ng mL^−1^)	Amount Detected (ng mL^−1^)	RSD (%)	Recovery (%)	Average Recovery (%)
1	5	5.89, 5.05, 5.24	8.17	117.8, 101.0, 104.8	107.5
2	10	9.29, 11.32, 10.49	9.85	92.9, 113.2, 104.9	
3	20	22.54, 23.50, 20.45	7.04	112.7, 117.5, 102.3	

**Table 2:** Comparison with other aptamer-sensing systems for CRP detections.

Technique	Linear Range (ng mL^−1^)	LOD (ng mL^−1^)	Ref.
SPR	5 × 10^−6^–5 × 10^−3^	5 × 10^−6^	[61]
Fluorescence	0.01–500	10^−2^	[62]
FET label free	12.5–8000	12.5	[63]
Electrochemical (label-free)	10^−3^–100	2.941 × 10^−4^	This work

## Data Availability

Not applicable.

## Supplementary Materials

The following supporting information can be downloaded at: https://www.mdpi.com/article/10.3390/bios12121180/s1, Figure S1: Voltage-current diagram of stability on day 1, day 3, day 5 and day 9; Figure S2: Voltage-current diagram of the reproducibility of five parallel electrodes.

## References

[1] Tillett W.S., Francis T. (1930). Serological Reactions in Pneumonia with a Non-Protein Somatic Fraction of Pneumococcus. J. Exp. Med..

[2] Sproston N.R., Ashworth J.J. (2018). Role of C-Reactive Protein at Sites of Inflammation and Infection. Front. Immunol..

[3] Frontera J.A., Provencio J.J., Sehba F.A., McIntyre T.M., Nowacki A.S., Gordon E., Weimer J.M., Aledort L. (2017). The Role of Platelet Activation and Inflammation in Early Brain Injury Following Subarachnoid Hemorrhage. Neurocrit. Care.

[4] Haider Kazmi S.J., Zafar M.T., Zia B.F., Khalid S.R., Kumar V., Tabassum S., Ali A., Aziz N., Khan N.A., Kumari K. (2022). Role of serum C-reactive protein (CRP)/Albumin ratio in predicting the severity of acute pancreatitis: A retrospective cohort. Ann. Med. Surg..

[5] Brull D.J., Serrano N., Zito F., Jones L., Montgomery H.E., Rumley A., Sharma P., Lowe G.D.O., World M.J., Humphries S.E. (2003). Human CRP Gene Polymorphism Influences CRP Levels. Arterioscler. Thromb. Vasc. Biol..

[6] Shah T., Casas J.P., Cooper J.A., Tzoulaki I., Sofat R., McCormack V., Smeeth L., Deanfield J.E., Lowe G.D., Rumley A. (2009). Critical appraisal of CRP measurement for the prediction of coronary heart disease events: New data and systematic review of 31 prospective cohorts. Int. J. Epidemiol..

[7] Mazer S.P., Rabbani L.E. (2004). Evidence for C-Reactive Protein’s Role in (CRP) Vascular Disease: Atherothrombosis, Immuno-Regulation and CRP. J. Thromb. Thrombolysis.

[8] Karakas M., Koenig W. (2009). CRP in Cardiovascular Disease. Herz.

[9] Fleischmann R.M., van der Heijde D., Gardiner P.V., Szumski A., Marshall L., Bananis E. (2017). DAS28-CRP and DAS28-ESR cut-offs for high disease activity in rheumatoid arthritis are not interchangeable. RMD Open.

[10] Allin K.H., Bojesen S.E., Nordestgaard B.G. (2009). Baseline C-Reactive Protein Is Associated with Incident Cancer and Survival in Patients with Cancer. J. Clin. Oncol..

[11] Li Y., Zhong X., Cheng G., Zhao C., Zhang L., Hong Y., Wan Q., He R., Wang Z. (2017). Hs-CRP and all-cause, cardiovascular, and cancer mortality risk: A meta-analysis. Atherosclerosis.

[12] Marnell L., Mold C., Du Clos T.W. (2005). C-reactive protein: Ligands, receptors and role in inflammation. Clin. Immunol..

[13] Karlović D., Serretti A., Vrkić N., Martinac M., Marčinko D. (2012). Serum concentrations of CRP, IL-6, TNF-α and cortisol in major depressive disorder with melancholic or atypical features. Psychiatry Res..

[14] Jha M.K., Minhajuddin A., Chin-Fatt C., Greer T.L., Carmody T.J., Trivedi M.H. (2019). Sex differences in the association of baseline c-reactive protein (CRP) and acute-phase treatment outcomes in major depressive disorder: Findings from the EMBARC study. J. Psychiatr. Res..

[15] Köhler-Forsberg O., Buttenschøn H.N., Tansey K.E., Maier W., Hauser J., Dernovsek M.Z., Henigsberg N., Souery D., Farmer A., Rietschel M. (2017). Association between C-reactive protein (CRP) with depression symptom severity and specific depressive symptoms in major depression. Brain Behav. Immun..

[16] Kuo H.-K., Yen C.-J., Chang C.-H., Kuo C.-K., Chen J.-H., Sorond F. (2005). Relation of C-reactive protein to stroke, cognitive disorders, and depression in the general population: Systematic review and meta-analysis. Lancet Neurol..

[17] Osimo E.F., Baxter L.J., Lewis G., Jones P.B., Khandaker G.M. (2019). Prevalence of low-grade inflammation in depression: A systematic review and meta-analysis of CRP levels. Psychol. Med..

[18] Chen W., Zheng K.I., Liu S., Yan Z., Xu C., Qiao Z. (2020). Plasma CRP level is positively associated with the severity of COVID-19. Ann. Clin. Microbiol. Antimicrob..

[19] Wang L. (2020). C-reactive protein levels in the early stage of COVID-19. Med. Mal. Infect..

[20] Shang W., Dong J., Ren Y., Tian M., Li W., Hu J., Li Y. (2020). The value of clinical parameters in predicting the severity of COVID-19. J. Med. Virol..

[21] Ong D.S.Y., de Man S.J., Lindeboom F.A., Koeleman J.G.M. (2020). Comparison of diagnostic accuracies of rapid serological tests and ELISA to molecular diagnostics in patients with suspected coronavirus disease 2019 presenting to the hospital. Clin. Microbiol. Infect..

[22] Zhang C., Zhou W., Wang J., Zhang J., Zhang C. (2022). Investigation of the quantitative detection of serum Helicobacter pylori antibody in clinical laboratories in China. J. Clin. Lab. Anal..

[23] Waleed N. (2018). Evaluation of Complement Components (C3 and C4) in Diabetic Retinopathy patients. Res. J. Pharm. Technol..

[24] Carnell G.W., Trombetta C.M., Ferrara F., Montomoli E., Temperton N.J. (2021). Correlation of Influenza B Haemagglutination Inhibiton, Single-Radial Haemolysis and Pseudotype-Based Microneutralisation Assays for Immunogenicity Testing of Seasonal Vaccines. Vaccines.

[25] Lehmann R., Friess U., Häring H.-U., Voelter W., Liebich H., Beck A. (2003). Investigation of a capillary electrophoretic approach for direct quantification of apolipoprotein A-I in serum. Electrophoresis.

[26] Yang C., Zhang X., Wang S., Huo X., Wang J. (2021). Small intestinal bacterial overgrowth and evaluation of intestinal barrier function in patients with ulcerative colitis. Am. J. Transl. Res..

[27] Hosseini S., Vázquez-Villegas P., Rito-Palomares M., Martinez-Chapa S.O., Hosseini S., Vázquez-Villegas P., Rito-Palomares M., Martinez-Chapa S.O. (2018). Advantages, Disadvantages and Modifications of Conventional ELISA. Enzyme-Linked Immunosorbent Assay (ELISA): From A to Z.

[28] Ahmadalinezhad A., Chen A. (2011). High-performance electrochemical biosensor for the detection of total cholesterol. Biosens. Bioelectron..

[29] Ren Y., Deng H., Shen W., Gao Z. (2013). A Highly Sensitive and Selective Electrochemical Biosensor for Direct Detection of MicroRNAs in Serum. Anal. Chem..

[30] Kazemi S.H., Ghodsi E., Abdollahi S., Nadri S. (2016). Porous graphene oxide nanostructure as an excellent scaffold for label-free electrochemical biosensor: Detection of cardiac troponin I. Mater. Sci. Eng. C.

[31] Cesewski E., Johnson B.N. (2020). Electrochemical biosensors for pathogen detection. Biosens. Bioelectron..

[32] Buch M., Rishpon J. (2008). An Electrochemical Immunosensor for C-Reactive Protein Based on Multi-Walled Carbon Nanotube-Modified Electrodes. Electroanalysis.

[33] Dong S., Zhang D., Cui H., Huang T. (2019). ZnO/porous carbon composite from a mixed-ligand MOF for ultrasensitive electrochemical immunosensing of C-reactive protein. Sens. Actuators B Chem..

[34] Huang S., Liu Z., Yan Y., Chen J., Yang R., Huang Q., Jin M., Shui L. (2022). Triple signal-enhancing electrochemical aptasensor based on rhomboid dodecahedra carbonized-ZIF67 for ultrasensitive CRP detection. Biosens. Bioelectron..

[35] Diculescu V.C., Chiorcea-Paquim A.-M., Oliveira-Brett A.M. (2016). Applications of a DNA-electrochemical biosensor. TrAC Trends Anal. Chem..

[36] Idili A., Parolo C., Ortega G., Plaxco K.W. (2019). Calibration-Free Measurement of Phenylalanine Levels in the Blood Using an Electrochemical Aptamer-Based Sensor Suitable for Point-of-Care Applications. ACS Sens..

[37] Idili A., Parolo C., Alvarez-Diduk R., Merkoçi A. (2021). Rapid and Efficient Detection of the SARS-CoV-2 Spike Protein Using an Electrochemical Aptamer-Based Sensor. ACS Sens..

[38] Chung S., Moon J.-M., Choi J., Hwang H., Shim Y.-B. (2018). Magnetic force assisted electrochemical sensor for the detection of thrombin with aptamer-antibody sandwich formation. Biosens. Bioelectron..

[39] Jarczewska M., Rębiś J., Górski Ł., Malinowska E. (2018). Development of DNA aptamer-based sensor for electrochemical detection of C-reactive protein. Talanta.

[40] Yang X., Wang Y., Wang K., Wang Q., Wang P., Lin M., Chen N., Tan Y. (2014). DNA aptamer-based surface plasmon resonance sensing of human C-reactive protein. RSC Adv..

[41] Liao Z., Zhang Y., Li Y., Miao Y., Gao S., Lin F., Deng Y., Geng L. (2019). Microfluidic chip coupled with optical biosensors for simultaneous detection of multiple analytes: A review. Biosens. Bioelectron..

[42] Tang M.-Q., Xie J., Rao L.-M., Kan Y.-J., Luo P., Qing L.-S. (2022). Advances in aptamer-based sensing assays for C-reactive protein. Anal. Bioanal. Chem..

[43] Zhang Y., Noji H. (2017). Digital Bioassays: Theory, Applications, and Perspectives. Anal. Chem..

[44] Shan C., Yang H., Han D., Zhang Q., Ivaska A., Niu L. (2010). Graphene/AuNPs/chitosan nanocomposites film for glucose biosensing. Biosens. Bioelectron..

[45] António M., Nogueira J., Vitorino R., Daniel-da-Silva A.L. (2018). Functionalized Gold Nanoparticles for the Detection of C-Reactive Protein. Nanomaterials.

[46] Pingarrón J.M., Yáñez-Sedeño P., González-Cortés A. (2008). Gold nanoparticle-based electrochemical biosensors. Electrochim. Acta.

[47] Yáñez-Sedeño P., Pingarrón J.M. (2005). Gold nanoparticle-based electrochemical biosensors. Anal. Bioanal. Chem..

[48] Solhi E., Hasanzadeh M., Babaie P. (2020). Electrochemical paper-based analytical devices (ePADs) toward biosensing: Recent advances and challenges in bioanalysis. Anal. Methods.

[49] Lawal A.T. (2019). Graphene-based nano composites and their applications. A review. Biosens. Bioelectron..

[50] Service R.F. (2009). Carbon Sheets an Atom Thick Give Rise to Graphene Dreams. Science.

[51] Li F., Long L., Weng Y. (2020). A Review on the Contemporary Development of Composite Materials Comprising Graphene/Graphene Derivatives. Adv. Mater. Sci. Eng..

[52] Szabó T., Berkesi O., Dékány I. (2005). DRIFT study of deuterium-exchanged graphite oxide. Carbon.

[53] Stankovich S., Piner R.D., Nguyen S.T., Ruoff R.S. (2006). Synthesis and exfoliation of isocyanate-treated graphene oxide nanoplatelets. Carbon.

[54] Li J., Liu S., Yu J., Lian W., Cui M., Xu W., Huang J. (2013). Electrochemical immunosensor based on graphene–polyaniline composites and carboxylated graphene oxide for estradiol detection. Sens. Actuators B Chem..

[55] Upan J., Youngvises N., Tuantranont A., Karuwan C., Banet P., Aubert P.-H., Jakmunee J. (2021). A simple label-free electrochemical sensor for sensitive detection of alpha-fetoprotein based on specific aptamer immobilized platinum nanoparticles/carboxylated-graphene oxide. Sci. Rep..

[56] Bharti A., Agnihotri N., Prabhakar N. (2019). A voltammetric hybridization assay for microRNA-21 using carboxylated graphene oxide decorated with gold-platinum bimetallic nanoparticles. Microchim. Acta.

[57] Ahmad H.M.N., Dutta G., Csoros J., Si B., Yang R., Halpern J.M., Seitz W.R., Song E. (2021). Stimuli-Responsive Templated Polymer as a Target Receptor for a Conformation-Based Electrochemical Sensing Platform. ACS Appl. Polym. Mater..

[58] Xiao Y., Lubin A.A., Heeger A.J., Plaxco K.W. (2005). Label-free electronic detection of thrombin in blood serum by using an aptamer-based sensor. Angew. Chem..

[59] Zhao Y., Gosai A., Shrotriya P. (2019). Effect of receptor attachment on sensitivity of label free microcantilever based biosensor using malachite green aptamer. Sens. Actuators B Chem..

[60] Ishida T., Murayama T., Taketoshi A., Haruta M. (2020). Importance of Size and Contact Structure of Gold Nanoparticles for the Genesis of Unique Catalytic Processes. Chem. Rev..

[61] Vance S.A., Sandros M.G.J.S.r. (2014). Zeptomole detection of C-reactive protein in serum by a nanoparticle amplified surface plasmon resonance imaging aptasensor. Sci. Rep..

[62] Liu Z., Luo D., Ren F., Ran F., Chen W., Zhang B., Wang C., Chen H., Wei J., Chen Q.J.R.a. (2019). Ultrasensitive fluorescent aptasensor for CRP detection based on the RNase H assisted DNA recycling signal amplification strategy. RSC Adv..

[63] Lee W.-B., Chen Y.-H., Lin H.-I., Shiesh S.-C., Lee G.-B.J.S., Chemical A.B. (2011). An integrated microfluidic system for fast, automatic detection of C-reactive protein. Sens. Actuators B Chem..

