# Supplementary material for: A Simple Label-Free Aptamer-Based Electrochemical Biosensor for the Sensitive Detection of C-Reactive Proteins
Source: Biosensors (Basel). 2022 Dec 18;12(12):1180. doi: 10.3390/bios12121180 (PMC9775280; doi:10.3390/bios12121180)
Supplement: Supplementary file 1 [file biosensors-12-01180-s001.zip › biosensors-2040160-supplementary.pdf]

biosensors

Supporting Information

A Simple Label-Free Aptamer-Based Electrochemical Biosensor for the Sensitive Detection of C-Reactive Proteins

Huilin Gao [1], Yongchang Bai [1], Baixun He [1] and Cherie S. Tan [1,2,*]

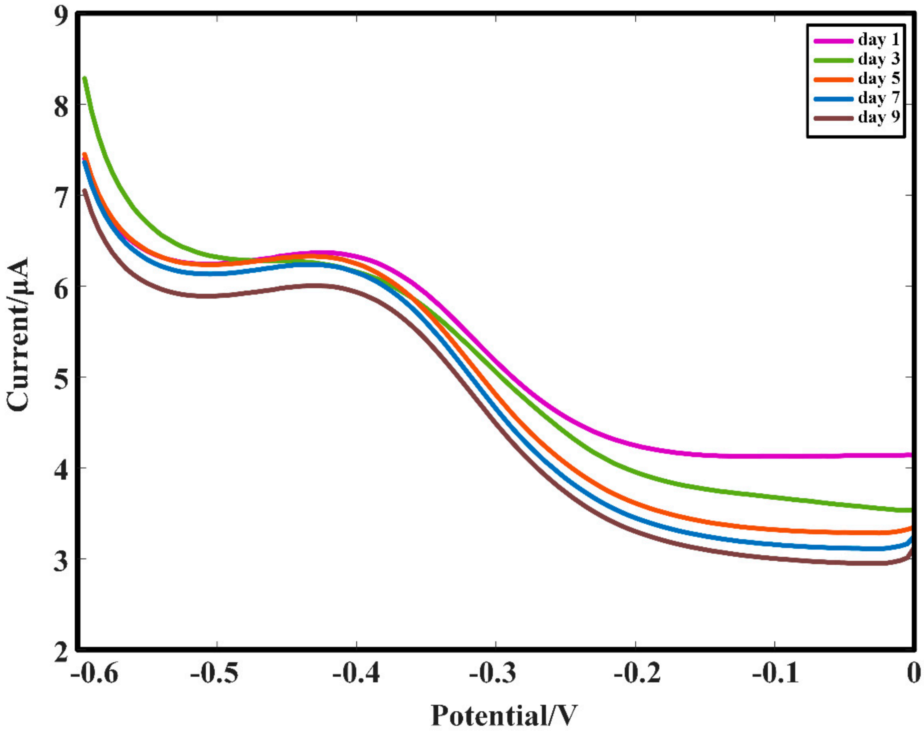

Figure S1. Voltage-current diagram of stability on day 1, day 3, day 5 and day 9.

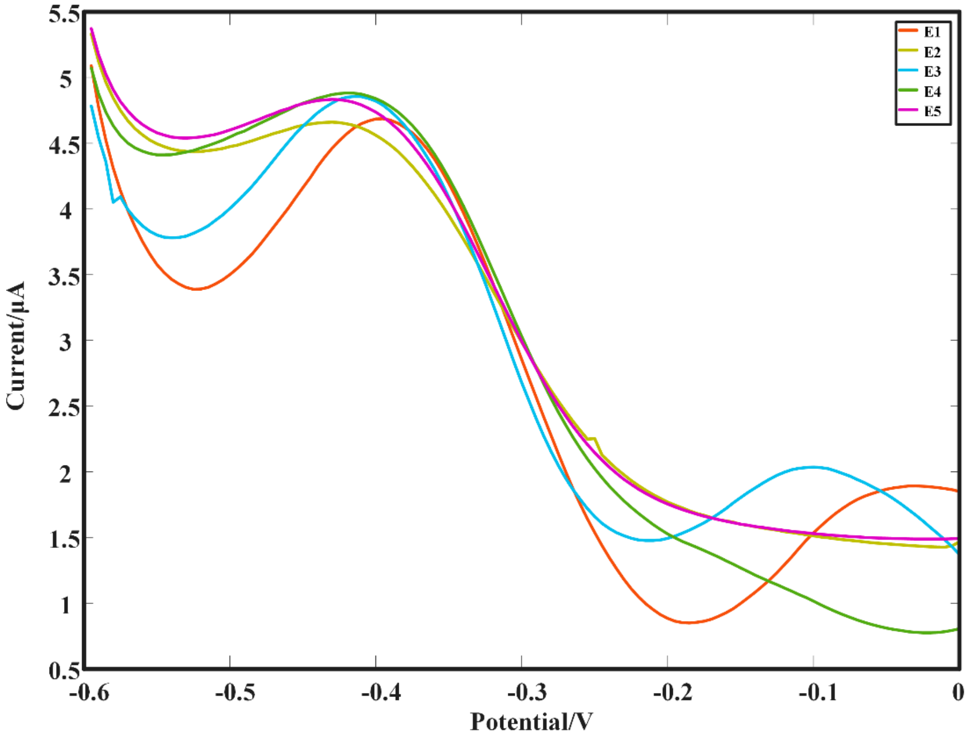

Figure S2. Voltage-current diagram of the reproducibility of five parallel electrodes.
